# Syncope After a Run

**DOI:** 10.1016/j.acepjo.2024.100038

**Published:** 2025-01-13

**Authors:** Olivia Bowles, Daniel Natkiel, Jeffrey Gardecki

**Affiliations:** 1 Department of Emergency Medicine, Jefferson Einstein Montgomery Hospital, East Norriton, Pennsylvania, USA; 2 Department of Emergency Medicine, Jefferson Einstein Philadelphia Hospital, Philadelphia, Pennsylvania, USA

**Keywords:** giant coronary artery aneurysm, RCA aneurysm, focused cardiac ultrasonography, syncope

## 1 Patient Presentation

A 66-year-old male with a history of hypertension presented to the emergency department (ED) following a syncopal episode. He reported 2 weeks of progressive fatigue and exercise intolerance. The episode occurred shortly after stopping a run on the treadmill. An elevated troponin led to discussion with cardiology and performance of a focused echocardiography (Video, Fig).

**Video:**
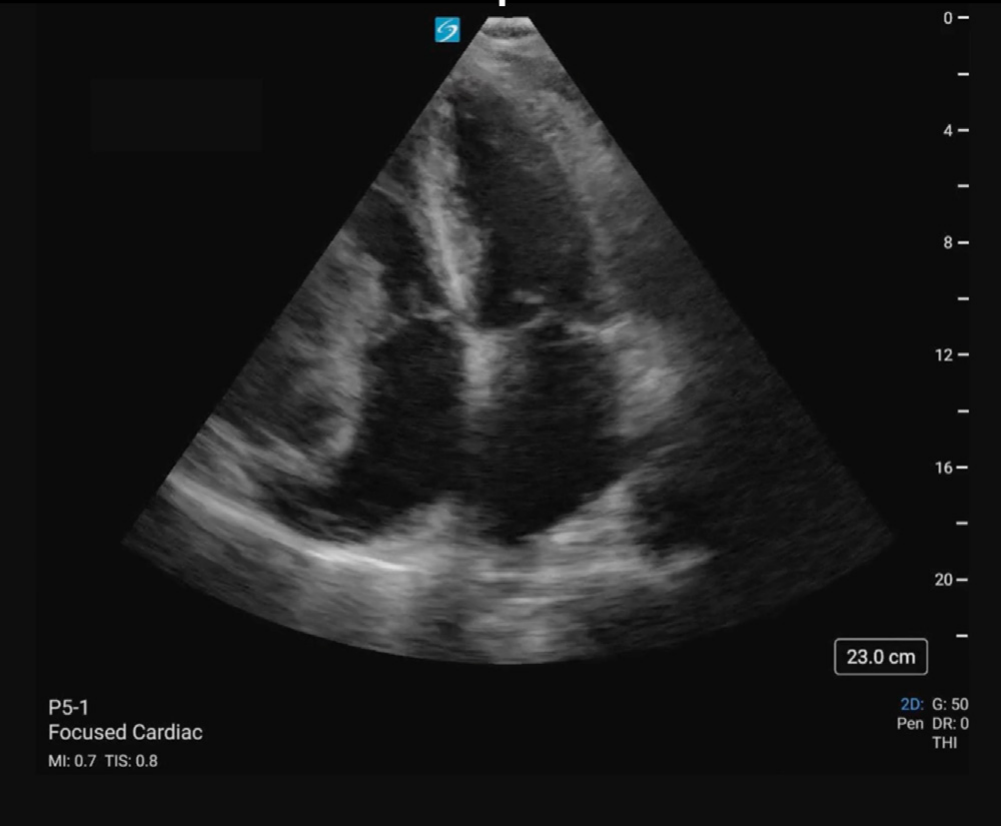
Apical 4-chamber view of the heart demonstrating a right heart mass with associated right ventricle free wall hypokinesis and small pericardial effusion.

**Figure:**
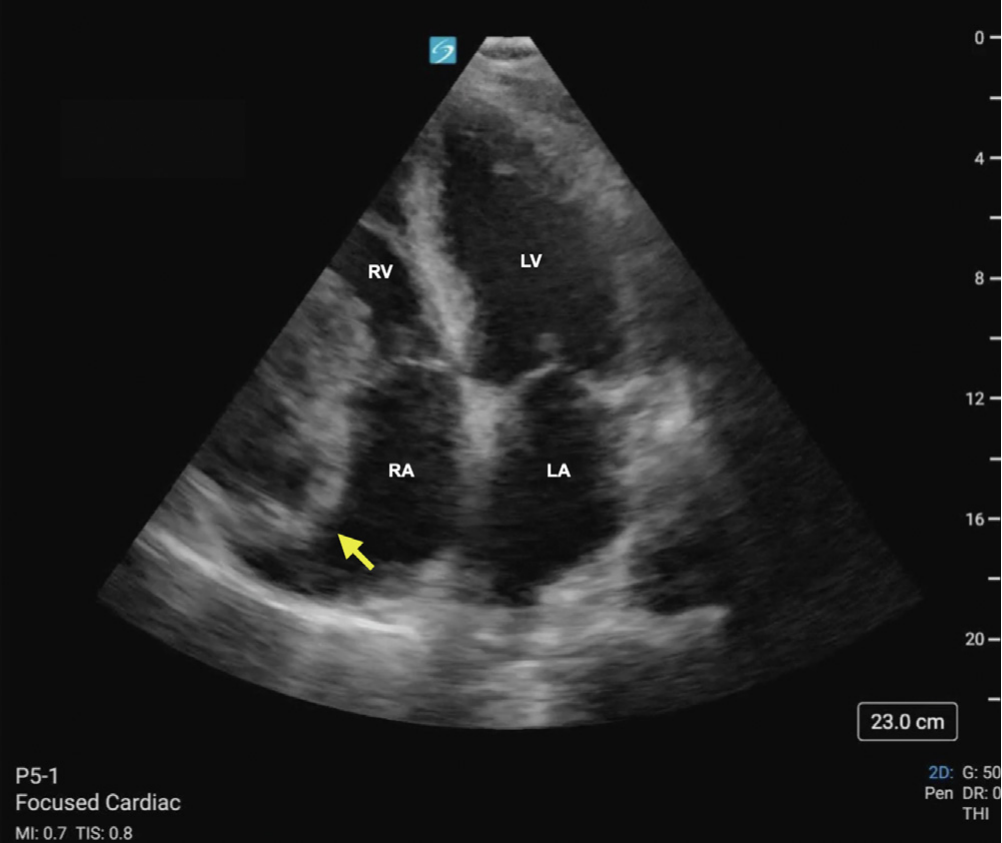
Still image of apical 4-chamber view of the heart with labels denoting cardiac chambers. The arrow denotes the right heart mass compressing the right atrium (RA) and right ventricle (RV). LA, left atrium; LV, left ventricle.

## 2 Diagnosis: Thrombosed Giant Right Coronary Artery Aneurysm With Contained Rupture, Right Coronary Artery to Right Ventricle Fistula

A cystic appearing mass can be seen abutting the right atrioventricular junction. Mass effect can be seen on the right atrium and ventricle (Video, Fig). Following discussion with cardiothoracic surgery, the patient ultimately underwent surgical repair of a right coronary artery (RCA) aneurysm and closure of a right ventricle to RCA fistula. Coronary artery aneurysms (CCAs) are defined as a dilation of the artery by 50% compared with its surrounding segments.^1^^,^^2^ CCAs are seen in 0.3% to 5% of patients undergoing coronary angiography.^1^^,^^2^ A giant CCA is a rarer entity with reported incidences of 0.02% in the general population. Giant CCAs often present symptomatically with chest pain or dyspnea and may be complicated by thrombus formation, embolization, or fistula formation.^3^ Although coronary angiography remains the gold standard for diagnosis, transthoracic or transesophageal echocardiography offers high sensitivity and specificity for evaluation of proximal right and left anterior descending coronary arteries.^3^ Treatment considerations are based on location, size, and presence of complications.^3^

## Funding and Support

By *JACEP Open* policy, all authors are required to disclose any and all commercial, financial, and other relationships in any way related to the subject of this article as per ICMJE conflict of interest guidelines (see www.icmje.org). The authors have stated that no such relationships exist.

## Conflict of Interest

All authors have affirmed they have no conflicts of interest to declare.
